# Evolutionary Pattern and Regulation Analysis to Support Why Diversity Functions Existed within PPAR Gene Family Members

**DOI:** 10.1155/2015/613910

**Published:** 2015-04-15

**Authors:** Tianyu Zhou, Xiping Yan, Guosong Wang, Hehe Liu, Xiang Gan, Tao Zhang, Jiwen Wang, Liang Li

**Affiliations:** ^1^Key Lab of Sichuan Province, Institute of Animal Genetics and Breeding, Sichuan Agricultural University, Ya'an, Sichuan 625014, China; ^2^College of Animal Science and Technology, Sichuan Agricultural University, Ya'an, Sichuan 625014, China

## Abstract

Peroxisome proliferators-activated receptor (PPAR) gene family members exhibit distinct patterns of distribution in tissues and differ in functions. The purpose of this study is to investigate the evolutionary impacts on diversity functions of PPAR members and the regulatory differences on gene expression patterns. 63 homology sequences of PPAR genes from 31 species were collected and analyzed. The results showed that three isolated types of PPAR gene family may emerge from twice times of gene duplication events. The conserved domains of HOLI (ligand binding domain of hormone receptors) domain and ZnF_C4 (C4 zinc finger in nuclear in hormone receptors) are essential for keeping basic roles of PPAR gene family, and the variant domains of LCRs may be responsible for their divergence in functions. The positive selection sites in HOLI domain are benefit for PPARs to evolve towards diversity functions. The evolutionary variants in the promoter regions and 3′ UTR regions of PPARs result into differential transcription factors and miRNAs involved in regulating PPAR members, which may eventually affect their expressions and tissues distributions. These results indicate that gene duplication event, selection pressure on HOLI domain, and the variants on promoter and 3′ UTR are essential for PPARs evolution and diversity functions acquired.

## 1. Introduction

Peroxisome proliferators-activated receptors (PPARs) are transcription factors belonging to the ligand-activated nuclear receptor superfamily, which play key roles in regulating metabolism, inflammation, and immunity. In vertebrates, the gene family of PPAR consisted of PPAR*α*, PPAR*β* (also called PPARb/d or PPAR*δ*), and PPAR*γ* [1]. Recently, a considerable number of papers have reviewed their importance in functions within various physiological and biochemistry processes [2–5]. Their special effects and functional manners of depending on a ligand-activated way even have attracted some scientists to consider them as a drug target for therapy of some metabolic disorders, such as the type 2 diabetes mellitus and atherosclerosis [6].

It has been well established that the PPARs can be divided into five distinct functional regions, which include DBD (DNA-binding domain), LBD (ligand-binding domain), AF1 (activation function 1), AF2 (activation function 2), and a variable hinge region. The DBD and LBD consist of a highly conserved DNA-binding domain and a moderately conserved ligand-binding domain, respectively. The AF1 and AF2 are two ligand-independent activation function domains. All these regions except the variable hinge region are highly conserved among PPAR members and are responsible for keeping their functions [3]. Although the PPARs share high similarities with each other in structures, they exhibit distinct patterns of distribution in tissues and differ in functions [7]. It has been summarized that PPAR*α* mainly is involved in the oxidation process of hepatocytes, PPAR*β* mainly targets within the adipocyte proliferation, and PPAR*γ* plays essential roles in origination and fate determination of preadipocyte. In adult rat, it has shown that PPARs had different expression patterns [8]. Definitely, PPAR*α* is highly expressed in hepatocytes, cardiomyocytes, enterocytes, and the proximal tubule cells of kidney, PPAR*β* is expressed ubiquitously and often at higher levels than PPAR*α* and PPAR*γ*, and PPAR*γ* is expressed predominantly in adipose tissue and the immune tissues [4].

It is interesting to investigate why PPARs exhibit distinct patterns of distribution in tissues and differ in functions even if they share high similarity of regions. There may be at least two main aspects of molecular reasons accounting for their differences. Firstly, it could be explained by the molecular evolutionary process, for example, the gene duplication event and the selective patterns. PPAR gene family as one of the nuclear hormone receptor (NHR) superfamilies evolves together with other NHR members. It has been demonstrated that a large number of NHR members are likely to result from two waves of gene duplication events. The first wave occurs before the arthropod/vertebrate divergence and has generated the ancestors of the NHR subfamilies, for instance, PPARs, RARs, and RXRs. The second wave of duplication is vertebrate-specific and leads to a diversification inside the subfamilies, with the emergence of the presently known isotypes such as PPAR*α*, PPAR*β*, and PPAR*γ* [3, 7]. However, it is still unknown which one is the common ancestor gene in PPAR members, and what the impacts of PPARs divergence on their functions are. Secondly, the special transcriptions factors binging in the promoter regions and the miRNAs target at 3′ UTRs of PPARs may be responsible for the distinct patterns of distribution in tissues. Numerous reports have established the basis for gene expression patterns in distribution by predicting and comprising the transcription factors and miRNAs of interested genes [9].

Therefore, in this present study, we took advantage of the availability of gene sequence data to analyze the PPAR gene family based on a view of molecular evolutionary relationship by deducing the possibility of evolution in PPAR gene family, as well as by predicting and comparing their transcription factors and miRNAs to primarily understand the reasons for diversity functions and distinct patterns of expressions in tissues of PPAR members. These analyses may contribute to a comprehensive understanding for the functions of PPAR gene family.

## 2. Materials and Methods

### 2.1. PPAR Gene Homology Sequence Collection

The Genomic Blast function (http://blast.ncbi.nlm.nih.gov/Blast.cgi) was used for collecting homologous sequences of PPAR gene family members in species. The parameters were set as the default value. For the minority of the PPAR gene sequences unfound by blast, we separated supplement in the website of Nucleotide database (http://www.ncbi.nlm.nih.gov/nuccore/) by manually using keywords. Through blasting the homology sequences of PPAR*α*, PPAR*β*, and PPAR*γ* on NCBI, we finally obtained 63 homology sequences that belong to 31 species (Table S1 in Supplementary Material available online at http://dx.doi.org/10.1155/2015/613910). Most of these sequences were from mammals, and a few of them were obtained from fish and birds. These collected sequences were edited and aligned by the MegAlign in DNAStar (Madison, Wisconsin, USA).

### 2.2. Search for Protein Domains

The open reading frames (ORF) of PPAR sequences in different species were predicted using online software (http://www.ncbi.nlm.nih.gov/gorf/orfig.cgi). Next, these ORF sequences were confirmed by Pfam (http://pfam.sanger.ac.uk/). Only if there were homology amino acid sequences blasted, Pfam would show the ORF sequences being correctly predicted. Furthermore, the correct amino acid sequences were entered into SMART (http://smart.embl-heidelberg.de/) platform for a prediction of protein structure domain.

### 2.3. Construction of Phylogenetic Tree

The format of each PPAR homologous protein sequence was edited by BioEdit software [10]. Then, the protein sequences were used for constructing phylogenetic tree through a model of maximum likelihood method (ML) by Mega 5.1 [11]. The topological stability of the maximum likelihood tree was evaluated by 1000 bootstrap replications. The Atlantic salmon PPAR*γ* protein sequence (NM_001123546.1) was selected as the outgroup of the protein phylogenetic tree.

### 2.4. Amino Acid Site Selection Pressure Analysis

The sequences of two conserved protein domains (ZnF_C4 and HOLI domains) were chosen and compared by BioEdit, and then they were classified and merged. According to the analysis of Bayesian tree phylogeny, we used the site model in PAML software package in Codeml program [12] to analyze these two domains.

The site model was constructed to test whether PPAR gene is subjected to positive selection (*ω* > 1) or negative selection (*ω* < 1) [13]. This model allows different sites to have different selection pressure, while there is no difference in different branches of the phylogenetic tree. The models named M1a (neutral) and M2a (selection) [13, 14] in the current study were used twice the log-likelihood difference (2Δ*L*) following *χ* ^2^ distribution of likelihood ratio test (LRT), the difference degree of freedom for the two parameters of the model number.

### 2.5. Analysis of Transcription Factors

By using Gene (http://www.ncbi.nlm.nih.gov/gene/) of the NCBI, the location of the PPAR gene was determined on the chromosome corresponding species. And then, we confirmed the first exon of the PPAR gene transcription initiation site on a chromosome. Sequence about 1000 bp was selected to use as the predicted promoter regions from the upstream of the first exon. On the TRANSFAC, the Alibaba (http://www.gene-regulation.com/pub/programs/alibaba2/index.html) can estimate transcription factor binding sites (TFBS) in unknown DNA sequences.

### 2.6. Predictions of miRNAs in 3′ UTR Region of PPAR Members

The miRNAs in 3′ UTR region of PPAR members and their regulatory sites were predicted by TargetScan release (http://www.targetscan.org/). In the TargetScan, the 3′ UTR region of PPAR members of human was searched for miRNAs. The search results were sorted in the miR2Disease Base (http://www.mir2disease.org/) for predicting functions of the predicted conservative miRNAs.

## 3. Results and Analysis

### 3.1. The Unique Homology and Conserved Domains in PPAR Gene Family

As it was shown in Table S1, the coding regions of all PPAR nucleotides were in average length of about 1400 bp, which encode about 466 amino acids. The average length of nucleotides of PPAR*α* coding domain is 1406 bp, whereas the average length of nucleotides of PPAR*β* is 1284 bp which is lower than the average value of the entire PPAR family. The nucleotide of PPAR*γ* is 1479 bp which is obviously higher than the average value.

**Figure 1:**
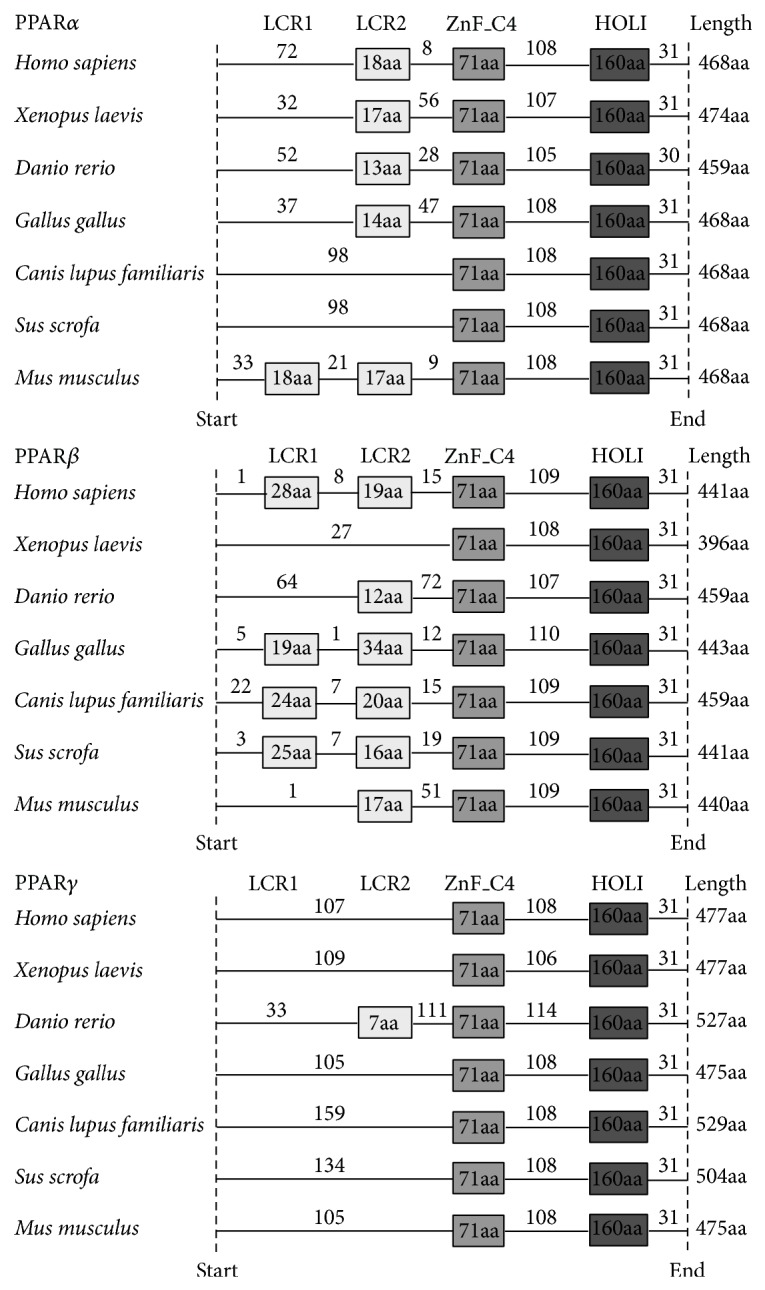
The protein domains of PPARs were predicted in 7 representative species. A box represents a conserved domain. The numerals labeled in the boxes and lines represent the number of amino acid residues. The PPARs coding domain sequences were collected in 7 representative species including human, xenopus, zebrafish, chicken, dog, pig, and mouse.

The protein domains were predicted corresponding to each sequence in the coding region through SMART. The PPAR coding domain sequences in 7 representative species including human, xenopus, zebrafish, chicken, dog, pig, and mouse were obtained for a further analysis (Figure 1). The data demonstrated that all PPARs family members contained the ZnF_C4 and HOLI domains, which are conserved among species. In addition to the conserved domains, low complexity 1 and low complexity 2 regions (LCRs) were in great differences among PPAR members and species. In PPAR*α*, it was found that LCR2 widely existed in most species, and LCR1 only existed in mice. It is also worth noticing that more than half of the studied species contained the LCRs domains in PPAR*β*, except for the absence of LCR2 in xenopus. In PPAR*γ*, the LCR2 domain was only found in zebrafish, whereas the LCR1 domain was absent in all studied species.

### 3.2. The Phylogenetic Tree of PPAR Gene Family

**Figure 2:**
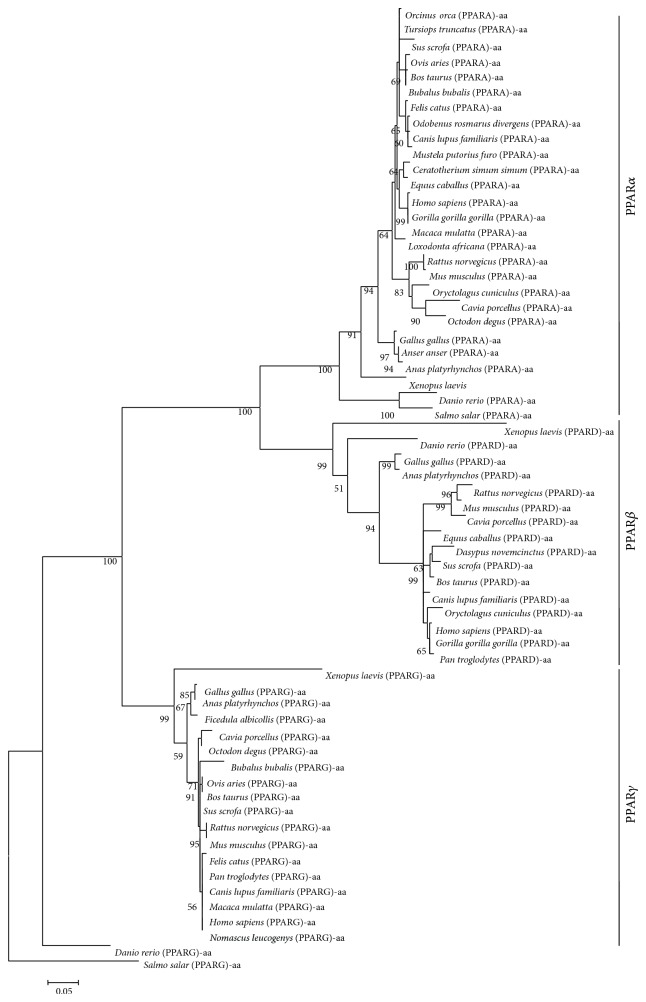
The phylogenetic tree based amino acid sequences. The phylogenetic tree was constructed by amino acid sequences. The sequences information was provided in Table S1. The phylogenetic tree was constructed by the maximum likelihood method with Mega 5.1. The numbers on nodes indicate the support values. It showed the bootstrap values were more than 50%.

In order to investigate the homologous relationships among PPAR gene family members, we constructed phylogenetic tree based on the amino acid level. The phylogenetic tree was constructed based on the 63 amino acid sequences from 31 species (Table S1), and the results were shown in Figure 2. The orthologs of PPAR members from fishes were placed at the base of the three branches of the tree. Furthermore, the PPAR genes were spitted into three lineages (support value = 100%). Through the branches and distances of the phylogenetic tree, PPAR*α* and PPAR*β* were clustered together. The branch of PPAR*γ* stood alone and was closer to the outgroup than the other two branches. PPAR*γ* might be the earliest ancestor form of the PPAR gene family. According to the classification, it suggested that the first independent duplication event may occur in bony fishes before separation from the birds and mammals during the whole evolutionary process of PPAR gene family. And after a second duplication event, the isolated types of PPAR*α* and PPAR*β* may emerge as the paralogs of PPAR*γ*.

### 3.3. Selection Pressure of Amino Acid Residues in PPAR Gene

To determine the selection states of each amino acid site in conserved structure of PPARs during the evolution process, the tools of selective pressure were used for investigating the different selection patterns based on the conserved motifs of ZnF_C4 domain and HOLI domain, which were widely included and conserved in PPAR gene family. In branch-site models (Table 1), we found the estimated *ω* value ≥ 1 with the M2a model for HOLI domain and ZnF_C4 domain. It suggests that PPAR genes were under positive selection. By the LRT test, M1a and M2a were compared with their corresponding null models (M0), respectively. The results suggested that M2a (*P* < 0.05) was more in coincidence with the data than M1a (*P* > 0.05). What is more, the LRT tests of all PPAR members were different. The HOLI domain could be accepted by M2a, indicating a positive selection pressure of HOLI domain during the molecular evolution process, whereas the ZnF_C4 domain was rejected.

**Figure 3:**
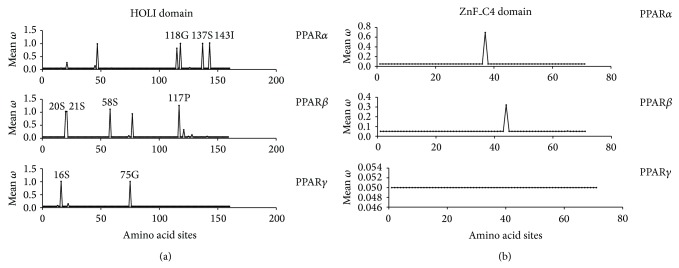
Approximate posterior mean of amino acid sites. There was a list of amino acids in each sequence of the corresponding *ω* value. The amino acid residue marked on the image represents the *ω* > 1 with probability of more than 95%.

In a 95% posterior probability, the results (Figures 3(a) and 3(b)) showed that the positive selection sites in PPAR*α* HOLI domain were 118G, 137S, and 143I, in PPAR*β* HOLI domain 20S, 21S, 58S, and 117P, and in PPAR*γ* HOLI domain 16S and 75G, whereas in the ZnF_C4 domain, there were no positive selection sites observed in all PPAR members, except for only one suspected amino acid residue with *ω* value between 0.5 and 1 observed in ZnF_C4 domain of PPAR*α* and PPAR*β*, respectively. In PPAR*γ* ZnF_C4 domain, there were no positive selection sites observed either.

### 3.4. Prediction of Transcription Factors

The transcription factors and their binding sites in promoter regions of PPAR gene family were predicted in human and chicken, respectively, and the results were listed in Table S2. In chicken, 45, 44, and 39 transcription factors were predicted and targeted at the promoter regions of PPAR*α*, PPAR*β*, and PPAR*γ*, respectively. In human, only a total of 31, 36, and 40 transcription factors have been predicted at promoter regions of PPAR*α*, PPAR*β*, and PPAR*γ*, respectively, which were different from it in chicken.

Through comparing transcription factors, we found that numerous common transcription factors existed among PPAR members. Then they were compared pairwise among the three PPAR members, and the results were listed in Table 2. The PPARs shared 9 common transcription factors which were targeted at the promoter regions, including Sp1, CPE_bind, CP1, Oct-1, GATA-1, AP-2*α*, NF-1, GR, and C/EBP*α* in human, while in chicken, 11 common transcription factors were predicted and targeted at the promoter regions of chicken PPARs, which included CREB, SRF, ICSBP, Ftz, AP-1, Oct-1, GATA-1, AP-2*α*, NF-1, GR, and C/EBP*α*. However, the binding sites for each common transcription factor were varied among PPAR members.

Finally, we quantified the coexisting transcription factors among PPAR members (Table 3). In human, the amount of the identical transcription factors between PPAR*α* and PPAR*β* was 18, while the amount between PPAR*β* and PPAR*γ* is 16. The number of identical transcription factors of PPAR*α* and PPAR*γ* was 12. In chicken, the group of PPAR*α*/*γ* and PPAR*α*/*β* shared 20 and 15 identical transcription factors, respectively.

### 3.5. Prediction of miRNAs Target at the 3′ UTR Region of PPAR Members

**Figure 4:**
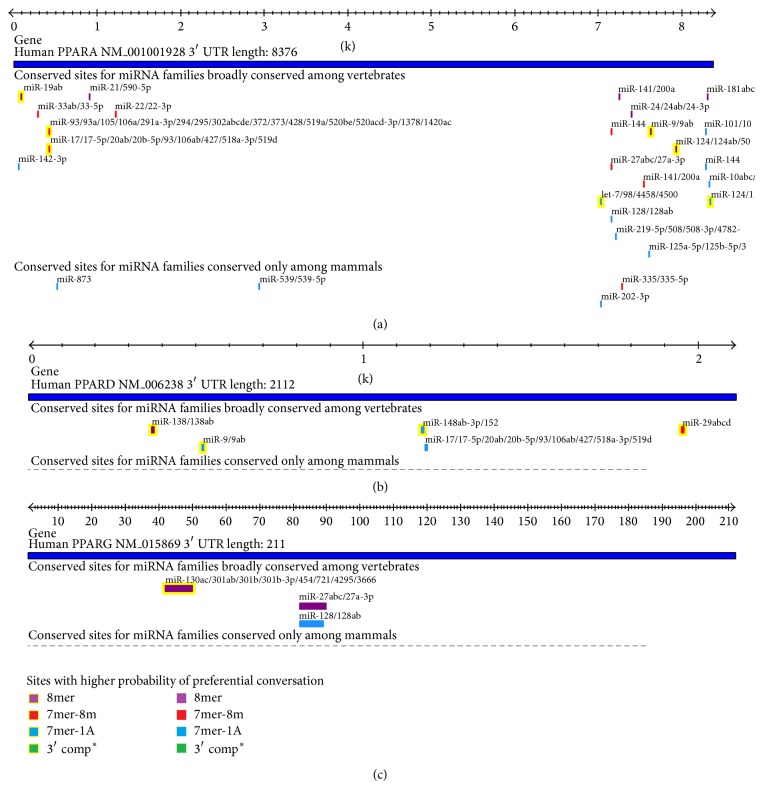
The miRNAs predicted and their targets sites in 3′ UTR region of PPAR genes in human. (a) PPAR*α*; (b) PPAR*β*; (c) PPAR*γ*. The miRNAs targets sites correspond to the 3′ UTR region of PPAR genes. The lower corner is the probability of preferential conservation for sites.

The miRNAs in 3′ UTR of PPAR members were predicted in human. The results (Table S3) showed that, in the 3′ UTR region of PPAR*α*, a total of 23 conserved binding sites of miRNAs were predicted in vertebrates, and 4 conserved sites of miRNA families were predicted in mammals. In the 3′ UTR region of PPAR*β* (Figure 4(b)) and PPAR*γ* (Figure 4(c)), 5 and 3 conserved sites of miRNA families were predicted in vertebrates, respectively. Notably, the miR-17 and miR-9 were predicted in both 3′ UTR regions of PPAR*α* and PPAR*β*, and the miR-27abc and miR-128 were predicted in both 3′ UTR regions of PPAR*α* and PPAR*γ* (Figure 4(a)).

The functions of these miRNAs were enriched in PUBMED online. Among the 27 miRNA families, the vast majority were closely related with cancer. For example, the miR-142-3p [15], miR-19a [16], and miR-124 [17] were reported to be involved in hepatocellular carcinoma; the miR-9 [18] targeting to the 3′ UTR region of PPAR*α* was associated with Hodgkin's lymphoma. In the 3′ UTR region of PPAR*β*, the miR-138 [19] were reported to be linked to anaplastic thyroid carcinoma; the miR-17 [20] was related to B-cell lymphoma; the miR-29c [21] was interrelated with chronic lymphocytic leukemia. In the 3′ UTR region of PPAR*γ*, the miR-128 [22] was associated with glioma.

## 4. Discussions

One new gene is mainly generated by the gene or genome duplication event [23]. PPARs as one of the NHR superfamilies evolve together with other NHR members, and after it has undergone twice time of gene duplication events, the vertebrate-specific PPAR is eventually diverged into three different isotypes [3, 7]. The phylogenetic tree of PPARs in the present study demonstrated that PPAR gene family may have yielded a gene duplication event, which first occurs in bony fishes before separation from the birds and mammals during the whole evolution process. PPAR*γ* is closer to the outgroup than the other two branches, supporting that PPAR*γ* might be the original ancestor gene in PPAR gene family. After being firstly duplicated in fish, PPAR begins to divide into two subtypes, including the PPAR*γ* and the common ancestor of PPAR*α* and PPAR*β*. These findings are consistent with the previous studies by Michalik et al., which depicted an evolutionary process of PPARs. Moreover, PPAR*α* and PPAR*β* were clustered closer than others, supporting that they may originate from a homology ancestor gene, and their divergence may result from another gene duplication event in vertebrates; however, there is no sufficient evidence to support this hypothesis currently.

Following the gene duplication event in PPARs, the newly emerging receptors would have acquired the ligand binding capacities in an independent fashion [24]. Once such capacity was acquired, each receptor of PPARs may begin to further evolve and refine its specificity for a given ligand. Each PPAR isotype may then evolve by mutations, which lead to a more specific range of ligands across species. These hypotheses could be supported by the sequence variants among PPARs across species in the present study. Our results showed that all PPAR members contained the conserved HOLI and ZnF_C4 domains, which are important for keeping the functions of PPAR gene family. HOLI domain located in N-terminal of the PPAR protein is also known as ligand binding domain of hormone receptors [25]. It belongs to the LBD region that acts in response to ligand binding, which caused a conformational change in the receptor to induce a response, thereby acting as a molecular switch to turn on transcriptional activity [26]. In addition, ZnF_C4 domain is also called C4 zinc finger in nuclear hormone receptors. This domain was the DBD region, which recognizes specific sequences, connected via a linker region to a C-terminal LBD. Both HOLI and ZnF_C4 domains are highly conserved among PPAR members and are responsible for keeping their basic functions for PPAR family members.

In addition to the two conserved domains, PPAR family contained low complexity regions (LCRs). LCRs located near the left of ZnF_C4 domain are in great differences among PPAR members across species. Studies suggested that the positions of LCRs within a sequence might be important to both determine their binding properties and maintain biological functions [27]. There are no LCRs existing in PPAR*γ*, suggesting that PPAR*γ* might only keep the basic function of PPAR family. The number of LCRs in PPAR*α* and PPAR*β* is similar and obviously more than PPAR*γ*, indicating differential functions of PPAR*α* and PPAR*β* from PPAR*γ*. The results showed that the variants in LCRs might be involved in the diversity functions of PPAR members and supported a common origin of PPAR*α* and PPAR*β*.

Due to the reason that ZnF_C4 and HOLI domain are important for keeping roles of PPAR members, we used patterns of selection pressure to analyze the adaptive evolution of the conserved protein sequences. The results showed that the HOLI domain was selected under a natural pressure in the evolutionary process, whereas the ZnF_C4 domain was not. It showed that ZnF_C4 domain was more conservative than HOLI domain in PPAR family, supporting a more important role of PPAR zinc finger in keeping PPARs' functions [28]. The HOLI domain in PPAR*β* with the most amounts of positive selection sites among PPAR members suggested that the variations in these positive selection sites were more beneficial for PPAR*β* phylogenetic towards diversity functions. Studies have confirmed that these chemical properties of amino acid residues were important to sustain normal protein folding and keep functions [29]. For instance, sulfhydryl groups of the peptide chain of two cysteines (cysteine, referred to as S) form two disulfide linkages with oxidation reaction. Whether it breaks or reshapes into a new one, it also could adjust protein to perform certain function [30]. Therefore, it can be inferred that the nucleotide variants in HOLI domain could be responsible for diversity functions of PPAR members. In a 95% posterior probability, the positive selection sites were 118G, 137S, and 143I in PPAR*α* HOLI domain, were 20S, 21S, 58S, and 117P in PPAR*β* HOLI domain, and were 16S and 75G in PPAR*γ* HOLI domain. It is interesting to point out that the positive selection sites in HOLI domain of PPAR*α* and PPAR*β* share more similarity in locations and amino acid residues, supporting a homology function of PPAR*α* and PPAR*β*.

The regulatory mechanism of gene expressions plays an important role in tissue distribution and distinct biological functions of genes. In eukaryotes, most genes are initiated and transcribed by lots of specific transcription factors targeting at their promoter regions [31]. Through predicting the transcription factors and their binding sites in promoter region of PPARs, we found that the transcription factors were varied among PPAR members in human and chicken, which may account for the specific tissue expression and distinct functions of PPARs. Some of these predicted transcription factors and their regulatory effects on PPARs are consistent with the previous reports; for example, the transcription factors AP1 and NF-kB were proved to enhance the expression of PPAR*β* activity [32]. Some of these transcription factors are also tissue specific, for example, the SP1 expressed in adenocarcinomas of the stomach [33], CP1 highly expressed in liver, kidney, and intestine but weakly expressed in adrenals and in lactating mammary glands [34, 35], and NF-1 detected in brain, peripheral nerve, lung, colon, and muscle [36], and so forth. It can be speculated that the variants in the promoter regions of PPAR*α* and PPAR*β* result into differential transcription factors binding on them that eventually influence their expressions and tissues distributions. Additionally, there are 18 common transcription factors between PPAR*β* and PPAR*α*, whereas the PPAR*γ* shared the least amount of common transcription factors with the other two members, which may contribute to the similarity in expression characteristics between PPAR*β* and PPAR*α*.

The miRNA can combine with the target mRNA by base pair, which leads to degradation or inhibition of the quantity levels of the target mRNA, thereby regulating gene expressions [37]. The regulation of miRNA on gene expressions is another path shaping gene expression patterns and biological processes [38]. In the present study, the miRNAs and their targets sites in 3′ UTR region of PPARs were predicted, and it was observed that the quantity of miRNAs was obviously differential in PPAR members. The number of miRNAs predicted in PPAR*α* was significantly more than the other two members. Moreover, it was worth noticing that most of the miRNAs were predicted in PPAR*α*, only a minority of them predicted in at least two PPAR isotypes; for example, only miRNA-128 was found in PPAR*α* and PPAR*γ* and miRNA-9 was found in PPAR*α* and PPAR*β*. These differences may be correlated with the distinct functions of PPAR isotypes, and PPAR*α* may be regulated by miRNAs in a much more complex way than the other two PPARs.

## 5. Conclusions

In the present study, the evolutionary pattern and regulation characteristics of PPARs were analyzed. The three isotypes of PPAR gene family may emerge from twice times of gene duplication events. PPAR*γ* might be the original ancestor gene in PPAR gene family. The conserved domains of HOLI domain and ZnF_C4 are essential for keeping basic roles of PPAR gene family, and the variant domain of LCRs may be responsible for their divergences in functions. The positive selection sites in HOLI domain are beneficial for PPARs to evolve towards diversity functions. The variants in the promoter regions and 3′ UTR of PPARs resulted into differential transcription factors and miRNAs involved in regulating PPAR members that may eventually influence their expressions and tissue distributions.

## Supplementary Material

Table S1: There are 27 sequences for PPAR*α*, 16 sequences for PPAR*β* and 20 sequences for PPAR*γ*. Each sequence represents a species and its accession number information.

## Figures and Tables

**Table 1:** Selection pressure analysis of amino acid sites in PPARs.

Model	ln*L*	Parameters estimates	2Δ*L*
Model 0			
*α*-HOLI	−4720.995579		
*β*-HOLI	−2960.312353		
*γ*-HOLI	−2719.120808		
*α*-ZnF_C4	−1050.374116		
*β*-ZnF_C4	−1033.465323		
*γ*-ZnF_C4	−1276.57302		
Model 1a			
*α*-HOLI	−4622.51301	*P*0 = 0.93383, *P*1 = 0.06617	
		*ω*0 = 0.01746, *ω*1 = 1.00000	
*β*-HOLI	−2894.97063	*P*0 = 0.94599, *P*1 = 0.05401	
		*ω*0 = 0.02167, *ω*1 = 1.00000	
*γ*-HOLI	−2689.49404	*P*0 = 0.97407, *P*1 = 0.02593	
		*ω*0 = 0.00539, *ω*1 = 1.00000	
*α*-ZnF_C4	−1062.29037	*P*0 = 0.98590, *P*1 = 0.01410	
		*ω*0 = 0.00862, *ω*1 = 1.00000	
*β*-ZnF_C4	−1027.39487	*P*0 = 0.97625, *P*1 = 0.02375	
		*ω*0 = 0.00722, *ω*1 = 1.00000	
*γ*-ZnF_C4	−1276.57373	*P*0 = 0.99999, *P*1 = 0.00001	
		*ω*0 = 0.00168, *ω*1 = 1.00000	
Model 2a			
*α*-HOLI	−4622.51301	*P*0 = 0.93383, *P*1 = 0.03245, *P*2 = 0.03372	196.96513
		*ω*0 = 0.01746, *ω*1 = 1.00000, *ω*2 = 1.00000	
*β*-HOLI	−2894.97063	*P*0 = 0.94599, *P*1 = 0.04003, *P*2 = 0.01398	130.683442
		*ω*0 = 0.02167, *ω*1 = 1.00000, *ω*2 = 1.00000	
*γ*-HOLI	−2689.49404	*P*0 = 0.97407, *P*1 = 0.00429, *P*2 = 0.02163	59.253532
		*ω*0 = 0.00539, *ω*1 = 1.00000, *ω*2 = 1.00000	
*α*-ZnF_C4	−1044.04256	*P*0 = 0.98590, *P*1 = 0.01410, *P*2 = 0.00000	12.66312
		*ω*0 = 0.00737, *ω*1 = 1.00000, *ω*2 = 6.14876	
*β*-ZnF_C4	−1027.39487	*P*0 = 0.97625, *P*1 = 0.00871, *P*2 = 0.01504	12.140902
		*ω*0 = 0.00722, *ω*1 = 1.00000, *ω*2 = 1.00000	
*γ*-ZnF_C4	−1276.57302	*P*0 = 1.00000, *P*1 = 0.00000, *P*2 = 0.00000	0.000004
		*ω*0 = 0.00168, *ω*1 = 1.00000, *ω*2 = 1.00000	

Note: selection pressure on amino acid sites of the inspection is based on the calculation of *dN*/*dS* (*ω*), where *dN* is nonsynonymous coding sequences of each base mutation rate (nonsynonymous substitution rate) and *dS* is a synonymous mutation rate (synonymous substitution rate). When the *ω* > 1, the gene is by positive selection; *ω* = 1, no selection pressure; *ω* < 1, by purifying selection.

**Table 2:** The common transcription factors predicted in human and in chicken.

Transcription factor	Binding sites and position					
	Chicken (*α*)	Human (*α*)	Chicken (*β*)	Human (*β*)	Chicken (*γ*)	Human (*γ*)
Oct-1	TTAT (−205)	TGCAT (−50)	TTAwTTk (−463)	GCTkT (−737)	AATAT (−18)	AATT (−75)
C/EBP*α*	TTGA (−62)	GTTGC (−302)	ACAT (−29)	ATCCCA (−23)	ACTC (−71)	TTGC (−192)
AP-2*α*	GGGG (−84)	GGCyG (−239)	GGCT (−108)	CCCrG (−65)	AGCCTG (−684)	GCCTG (−136)
NF-1	TTTTGG (−457)	TGGCCA (−127)	GCCAA (−140)	TGsC (−15)	TGCCA (−560)	GCCAA (−383)
GR	TGTTCT (−137)	ACAA (−185)	AGAACA (−26)	ACAsA (−123)	ACAG (−128)	AGAAC (−679)
GATA-1	TTAT (−205)	GsATT (−51)	GCAGA (−312)	CwGAT (−175)	AGATA (−58)	CTTATC (−438)
CREB	GTCA (−942)		CGTCA (−941)		ACrTCA (−432)	
SRF	GCCwT (−385)		TTCCGG (−896)		AnATGG (−174)	
ICSBP	GGAAA (−399)		CCCT (−39)		GTTT (−42)	
Ftz	TAAT (−840)		TTAATT (−463)		TAAwTG (−343)	
AP-1	TGAsT (−776)		TCAGC (−556)		TGACTC (−69)	
Sp1		GGAGGG (−12)		GrGG (−38)		TGGG (−139)
CPE_bind		CrTCA (−74)		TGACGT (−968)		CCCC (−876)
CP1		ATTGG (−125)		ATTGG (−913)		AkTGGT (−401)

**Table 3:** The number of identical transcription factors among PPARs in human and in chicken.

	Human			Chicken		
	PPAR*α*	PPAR*β*	PPAR*γ*	PPAR*α*	PPAR*β*	PPAR*γ*
PPAR*α*	—	18	12	—	15	20
PPAR*β*	18	—	16	15	—	18
PPAR*γ*	12	16	—	20	18	—

## References

[1] Tontonoz P., Hu E., Spiegelman B. M. (1994). Stimulation of adipogenesis in fibroblasts by PPAR*γ*2, a lipid-activated transcription factor. *Cell*.

[2] Clark R. B. (2002). The role of PPARs in inflammation and immunity. *Journal of Leukocyte Biology*.

[3] Daynes R. A., Jones D. C. (2002). Emerging roles of PPARs in inflammation and immunity. *Nature Reviews Immunology*.

[4] Shiue Y.-L., Chen L.-R., Tsai C.-J., Yeh C.-Y., Huang C.-T. (2013). Emerging roles of peroxisome proliferator-activated receptors in the pituitary gland in female reproduction. *Biomarkers and Genomic Medicine*.

[5] Bishop-Bailey D., Bystrom J. (2009). Emerging roles of peroxisome proliferator-activated receptor-*β*/*δ* in inflammation. *Pharmacology and Therapeutics*.

[6] Berger J. P., Akiyama T. E., Meinke P. T. (2005). PPARs: therapeutic targets for metabolic disease. *Trends in Pharmacological Sciences*.

[7] Michalik L., Desvergne B., Dreyer C., Gavillet M., Laurini R. N., Wahli W. (2002). PPAR expression and function during vertebrate development. *International Journal of Developmental Biology*.

[8] Braissant O., Foufelle F., Scotto C., Dauça M., Wahli W. (1996). Differential expression of peroxisome proliferator-activated receptors (PPARs): tissue distribution of PPAR-*α*, -*β*, and -*γ* in the adult rat. *Endocrinology*.

[9] Wu X., Zou X., Chang Q. (2013). The evolutionary pattern and the regulation of stearoyl-CoA desaturase genes. *BioMed Research International*.

[10] Hall T. A. (1999). BioEdit: a user-friendly biological sequence alignment editor and analysis program for Windows 95/98/NT. *Nucleic Acids Symposium Series*.

[11] Tamura K., Dudley J., Nei M., Kumar S. (2007). MEGA4: molecular evolutionary genetics analysis (MEGA) software version 4.0. *Molecular Biology and Evolution*.

[12] Yang Z. (2007). PAML 4: phylogenetic analysis by maximum likelihood. *Molecular Biology and Evolution*.

[13] Nielsen R., Yang Z. (1998). Likelihood models for detecting positively selected amino acid sites and applications to the HIV-1 envelope gene. *Genetics*.

[14] Yang Z., Swanson W. J., Vacquier V. D. (2000). Maximum-likelihood analysis of molecular adaptation in abalone sperm lysin reveals variable selective pressures among lineages and sites. *Molecular Biology and Evolution*.

[15] Gramantieri L., Ferracin M., Fornari F. (2007). Cyclin G1 is a target of miR-122a, a MicroRNA frequently down-regulated in human hepatocellular carcinoma. *Cancer Research*.

[16] Budhu A., Jia H. L., Forgues M. (2008). Identification of metastasis-related microRNAs in hepatocellular carcinoma. *Hepatology*.

[17] Furuta M., Kozaki K. I., Tanaka S., Arii S., Imoto I., Inazawa J. (2009). miR-124 and miR-203 are epigenetically silenced tumor-suppressive microRNAs in hepatocellular carcinoma. *Carcinogenesis*.

[18] Nie K., Gomez M., Landgraf P. (2008). MicroRNA-mediated down-regulation of PRDM1/Blimp-1 in Hodgkin/Reed-Sternberg cells: a potential pathogenetic lesion in Hodgkin lymphomas. *American Journal of Pathology*.

[19] Mitomo S., Maesawa C., Ogasawara S. (2008). Downregulation of miR-138 is associated with overexpression of human telomerase reverse transcriptase protein in human anaplastic thyroid carcinoma cell lines. *Cancer Science*.

[20] Inomata M., Tagawa H., Guo Y. M., Kameoka Y., Takahashi N., Sawada K. (2009). MicroRNA-17-92 down-regulates expression of distinct targets in different B-cell lymphoma subtypes. *Blood*.

[21] Calin G. A., Ferracin M., Cimmino A. (2005). A MicroRNA signature associated with prognosis and progression in chronic lymphocytic leukemia. *The New England Journal of Medicine*.

[22] Zhang Y., Chao T., Li R. (2009). MicroRNA-128 inhibits glioma cells proliferation by targeting transcription factor E2F3a. *Journal of Molecular Medicine*.

[23] Prince V. E., Pickett F. B. (2002). Splitting pairs: the diverging fates of duplicated genes. *Nature Reviews Genetics*.

[24] Escriva H., Bertrand S., Laudet V. (2004). The evolution of the nuclear receptor superfamily. *Essays in Biochemistry*.

[25] Berger J., Moller D. E. (2002). The mechanisms of action of PPARs. *Annual Review of Medicine*.

[26] Edwards D. P. (2000). The role of coactivators and corepressors in the biology and mechanism of action of steroid hormone receptors. *Journal of Mammary Gland Biology and Neoplasia*.

[27] Coletta A., Pinney J. W., Solís D. Y. W., Marsh J., Pettifer S. R., Attwood T. K. (2010). Low-complexity regions within protein sequences have position-dependent roles. *BMC Systems Biology*.

[28] Nolte R. T., Wisely G. B., Westin S. (1998). Ligand binding and co-activator assembly of the peroxisome proliferator- activated receptor-*γ*. *Nature*.

[29] Hogg P. J. (2003). Disulfide bonds as switches for protein function. *Trends in Biochemical Sciences*.

[30] Wedemeyer W. J., Welker E., Narayan M., Scheraga H. A. (2000). Disulfide bonds and protein folding. *Biochemistry*.

[31] Rahman I., MacNee W. (1998). Role of transcription factors in inflammatory lung diseases. *Thorax*.

[32] Delerive P., de Bosscher K., Besnard S. (1999). Peroxisome proliferator-activated receptor *α* negatively regulates the vascular inflammatory gene response by negative cross-talk with transcription factors NF-*κ*B and AP-1. *Journal of Biological Chemistry*.

[33] Infantino V., Convertini P., Iacobazzi F., Pisano I., Scarcia P., Iacobazzi V. (2011). Identification of a novel Sp1 splice variant as a strong transcriptional activator. *Biochemical and Biophysical Research Communications*.

[34] Schweifer N., Barlow D. P. (1996). The Lx1 gene maps to mouse Chromosome 17 and codes for a protein that is homologous to glucose and polyspecific transmembrane transporters. *Mammalian Genome*.

[35] Alnouti Y., Petrick J. S., Klaassen C. D. (2006). Tissue distribution and ontogeny of organic cation transporters in mice. *Drug Metabolism and Disposition*.

[36] Andersen L. B., Ballester R., Marchuk D. A. (1993). A conserved alternative splice in the von recklinghausen neurofibromatosis (NF1) gene produces two neurofibromin isoforms, both of which have GTPase-activating protein activity. *Molecular and Cellular Biology*.

[37] Lewis B. P., Burge C. B., Bartel D. P. (2005). Conserved seed pairing, often flanked by adenosines, indicates that thousands of human genes are microRNA targets. *Cell*.

[38] Appasani K. (2008). *MicroRNAs: From Basic Science to Disease Biology*.

