# Supplementary material for: Evolutionary Pattern and Regulation Analysis to Support Why Diversity Functions Existed within PPAR Gene Family Members
Source: Biomed Res Int. 2015 Apr 15;2015:613910. doi: 10.1155/2015/613910 (PMC4413253; doi:10.1155/2015/613910)
Supplement: Supplementary file 1 — Table S1: There are 27 sequences for PPARα, 16 sequences for PPARβ and 20 sequences for PPARγ. Each sequence represents a species and its accession number information. [file 613910.f1.pdf]

Table S1 Gene accession numbers in GenBank

Gene name	Specie name	GenBank GIEST	Nucleotide length	Protein length
PPARα	Homosapiens	Y07619.1	1407	468
	Macaca mulatta	NM_001033029.1	1404	467
	Gorilla gorilla gorilla	XM_004063652.1	9881	468
	Xenopus laevis	NM_001089813.1	3355	474
	Danio rerio	DQ839548.1	1380	459
	Salmo salar	NM_001123560.1	1644	464
	Odobenus rosmarus divergens	XM_004415835.1	2002	468
	Orcinus orca	XM_004279591.1	1932	469
	Tursiops truncatus	XM_004322234.1	1802	469
	Gallus	NM_001001464.1	2100	468
	Anser anser	AF481797.1	1455	468
	Anas platyrhynchos	EF534215.1	1430	468
	Felis catus	XM_003989394.1	1420	468
	Canis lupus familiaris	NM_001003093.1	2032	468
	Oryctolagus cuniculus	XM_002723354.1	1407	468
	Mustela putorius furo	XM_004813909.1	8279	468
	Equus caballus	NM_001242553.1	1763	468
	Ovis aries	XM_004007050.1	1445	470
	Bos taurus	NM_001034036.1	1979	470
	Bubalus bubalis	JQ070333.1	1548	470
	Sus scrofa	NM_001044526.1	1488	468
	Ceratotherium simum simum	XM_004437986.1	1803	467
	Loxodonta africana	XM_003420893.1	1407	468
	Mus musculus	BC016892.1	2336	468
	Rattus norvegicus	NM_013196.1	2022	468
	Cavia porcellus	NM_001173004.1	2073	467
	Octodon degus	XM_004642403.1	1404	467
PPARβ	Homosapiens	AY919140.1	1326	441
	Gorilla gorilla gorilla	XM_004043869.1	3454	343
	Pan troglodytes	XM_003311239.1	3425	402
	Xenopus laevis	NM_001087841.1	1537	396
	Danio rerio	NM_131468.2	3219	517
	Gallus	NM_204728.1	1756	443
	Anas platyrhynchos	XM_005023480.1	2268	345
	Canis lupus familiaris	NM_001048102.1	1864	459
	Oryctolagus cuniculus	NM_001082000.1	1326	441
	Dasypus novemcinctus	XM_004458309.1	2521	442
	Equus caballus	XM_001498870.3	1375	402
	Bos taurus	NM_001083636.1	3300	441
	Sus scrofa	GU565977.1	2717	441

	Mus musculus	U10375.1	1323	440
	Rattus norvegicus	U75918.1	1975	440
	Cavia porcellus	XM_003473900.2	3245	440
PPARγ	Homosapiens	X90563.1	1766	477
	Macaca mulatta	NM_001032860.1	1759	505
	Nomascus leucogenys	XM_004091756.1	1663	483
	Pan troglodytes	XM_003950030.1	1663	483
	Xenopus laevis	NM_001087843.1	2046	477
	Danio rerio	NM_131467.1	1994	527
	Salmo salar	NM_001123546.1	2528	544
	Gallus	NM_001001460.1	2089	475
	Anas platyrhynchos	EF546801.2	1650	475
	Ficedula albicollis	XM_005052821.1	4207	475
	Felis catus	NM_001113176.1	1841	529
	Canis lupus familiaris	NM_001024632.2	1830	529
	Ovis aries	NM_001100921.1	1428	475
	Bos taurus	BC116098.1	1801	475
	Bubalus bubalis	HQ270143.1	1624	505
	Sus scrofa	NM_214379.1	1758	504
	Mus musculus	U10374.1	1428	475
	Rattus norvegicus	Y12882.2	1570	505
	Cavia porcellus	XM_003462736.2	1431	475
	Octodon degus	XM_004645604.1	1449	475

Table S2 All of the predicted TFBS in human and in chicken

PPARα		PPARβ		PPARγ	
Homo sapiens	Gallus gallus	Homo sapiens	Gallus gallus	Homo sapiens	Gallus gallus
Sp1	Oct-1	NF-1	p40x	p40x	Oct-1
Oct-1	C/EBPα	C/EBPα	GR	Oct-1	ICSBP
GATA-1	TBP	Sp1	C/EBPα	Zen-1	GATA-1
CREB	AP-2α	AP-2α	ICSBP	TBP	Erg-1
CPE_bind	NF-1	GR	ISGF-3	MEB-1	C/EBPα
CP1	GR	Hb	REV-ErbA	Hb	c-Jun
NF-1	HSTF	p40x	Sp1	AP-2α	c-Fos
AP-2α	Pit-1a	AP-1	AP-2α	Sp1	Pap1+
GR	Sp1	GATA-1	USF	C/EBPα	AP-1
Egr-1	Hb	TBP	c-Fos	YY1	NF-1
WT1	GATA-1	Pit-1a	NF-1	HNF-3B	GR
CACCC-bi	SGF-1	HSE-bind	ACE2	REB1	HNF-3B
MyoD	HSE-bind	HSF	NF-E2	ER	SRF
C/EBPα	Pit-1	MBP-1_(1)	c-Jun	NF-1	Dl
GLI3	NF-A	NF-kappa	AP-1	Id3	TBP
ENKTF-1	Elk-1	NF-kappaB	ITF-2	HNF-1	MATα2
E1	GCN4	Kr	MRF4	NF-ATc3	Hb
ICSBP	Antp	ARP-1	Id2	USF	Dfd
Krox-20	SRY	ATF	NF-kappaB	CP1	MEB-1
ETF	CoS	Oct-1	GATA-1	GATA-1	GLO
MIG1	MEB-1	USF	COUP	LyF-1	ATF
RAR-α	GLO	CeMyoD	RAR-α	T3R	Oct-21
REV-ErbAα	CREB	YY1	RXR-α	ICSBP	Eve
CoS	PU.1	N-Myc	ER	RAP1	Ftz
NF-kappa	SRF	CP1	c-Ets-1	C/EBPδ	HNF-1C
Adf-1	ICSBP	CTF	SRF	TSF3	ER
CTF	HNF-3	ETF	PR	c-Ets-1_	PR
RAP1	NRL	MIG1	Ftz	GR	CREB
NF-muE1	NF-kappaB	c-Jun	Oct-1	PR	Pit-1
Elk-1	C/EBPβ	CRE-BP1	IRF-1	T3R-α	USF
SRF	SGF-3	CREB	REB1	IRF-1	E2
	NF-ATc3	CPE_bind	YY1	E1	Pit-1a
	RSRFC4	Adf-1	AP-2	REV-ErbA	AP-2α
	CTF	WT1	Egr-1	COUP	c-Rel
	Odd	NF-muE1	CPE_bind	c-Jun	TCF-1α
	CeMyoD	Krox-20	ETF	NF-kappaB	Antp
	PEA3		ATF	CPE_bind	ISGF-3
	Ftz		EFI	Dfd	HNF-3
	C/EBP		CP1	Ftz	NF-EM5

	CPC1		CREB	HNF-1C	
	AP-1		WT1		
	p40x		CRE-BP1		
	HNF-1C		Adf-1		
	CPE_bind		AhR		
	Zen-1				

Table S3 The predicted miRNAs targets in PPAR gene family in human

miRNA	Gene name
hsa-miR-128	PPARα, PPARγ
hsa-miR-27abc	PPARα, PPARγ
hsa-miR-454	PPARγ
hsa-miR-142-3p	PPARα
hsa-miR-9	PPARα, PPARβ
hsa-miR-17	PPARα, PPARβ
hsa-miR-152	PPARβ
hsa-miR-138	PPARβ
hsa-miR-29abcd	PPARβ
hsa-miR-873	PPARα
hsa-miR-21	PPARα
hsa-miR-22	PPARα
hsa-miR-539	PPARα
hsa-miR-202-3p	PPARα
hsa-let-7	PPARα
hsa-miR-93	PPARα
hsa-miR-144	PPARα
hsa-miR-19ab	PPARα
hsa-miR-508	PPARα
hsa-miR-141	PPARα
hsa-miR-335	PPARα
hsa-miR-24	PPARα
hsa-miR-33ab	PPARα
hsa-miR-125a-5p	PPARα
hsa-miR-124	PPARα
hsa-miR-181abcd	PPARα
hsa-miR-101	PPARα
hsa-miR-10abc	PPARα
